# Liposome‐Based Potential Vaccines Platforms that Are Noncytotoxic

**DOI:** 10.1002/open.202500530

**Published:** 2026-01-11

**Authors:** Saida Mebarek, Killian Jacob, Carmela Ilaria Pierro, Davide Romanini, Michele Fiore

**Affiliations:** ^1^ Institut de Chimie et Biochimie Moléculaires et Supramoléculaires, ICBMS, UMR 5246, CNRS Université de Lyon, Université Claude Bernard Lyon 1 Villeurbanne France; ^2^ Department of Chemistry University of Bari “Aldo Moro” Bari Italy; ^3^ Dipartimento di Farmacia Università degli studi di Salerno Via Giovanni Paolo II 132 Salerno Italy

**Keywords:** glycolipids, liposomes, nanomedicine, tn antigen, vaccine platform

## Abstract

Synthetic vaccines represent a promising avenue in cancer immunotherapy by promoting targeted immune responses. Liposomal technologies have further advanced synthetic vaccinology by enabling the efficient delivery of tumor‐associated carbohydrate antigens. Despite this progress, the toxicity and reproducibility of such platforms remain underexplored. In this preliminary study, we synthesized a series of neoglycolipids bearing the Thomsen–Nouveau (Tn) antigen using bio‐orthogonal thiol–ene click chemistry. Here we present the results obtained using a set of neoglycolipids that were evaluated for their ability to self‐assemble into liposomal vesicles and for in vitro cytotoxicity. The resulting neoglycolipids exhibited no detectable cytotoxicity and formed stable liposomal structures when formulated with palmitic acid and 1‐palmitoyl‐2‐oleoyl‐*sn*‐glycero‐3‐phosphocholine via a freeze–thaw/extrusion process. This early‐stage work presents a proof of concept for a tunable, liposomal‐based synthetic vaccine platform.

## Graphical abstract

Engineered neoglycolipids were formulated with palmitic acid and 1‐palmitoyl‐2‐oleoyl‐*sn*‐glycero‐3‐phosphocholine into stable, noncytotoxic liposomes using a freeze–thaw/extrusion method. This proof‐of‐concept study demonstrates a modular liposomal vaccine platform, offering a safe and tunable carrier for antigen delivery, with potential applications in cancer immunotherapy.

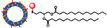

## 1 Introduction

Dendrimers [1], calixarenes [2], fullerenes [3], cyclodextrins [4], peptides [5, 6, 7, 8, 9], and immunogenic cyclopeptides [10, 11, 12, 13, 14, 15, 16, 17, 18] have been used in the last decades as platforms for anticancer drug delivery or self‐adjuvating vaccine cancer themself. Although encouraging, the outcomes achieved have not extended beyond the initial disclosure of preliminary biological data with some exceptions [6, 19]. The synthesis of the aforementioned glycoclusters typically involves considerable synthetic complexity, multiple iterative steps, often requiring repeated chromatographic purifications (e.g. high‐performance liquid chromatography), which can lead to low overall yields. In some cases, purification and reproducibility present significant bottlenecks [10, 18]. The application of chemoselective and bio‐orthogonal ligation strategies has been employed to partially address these limitations, owing to their operational simplicity and resistance to enzymatic degradation during conjugation processes [20]. Furthermore, the number of exposed antigens remains limited to the structure of the glycocluster [21] itself with the undesired clustering side effect [22]. Recent results showed that glycolipids assembled via thiol–ene click (TEC) chemistry (bearing two GlcNAc‐SH sugar moieties on the same scaffold) exhibited a binding affinity to wheat germ agglutinin 3000‐fold higher than that of the corresponding monosaccharide alone [23]. Recent advances in glycolipid synthesis have demonstrated significant potential for the fabrication of supramolecular assemblies that mimic the morphology and stability of membrane vesicles, ranging from micrometric (giant unilamellar vesicles, GUV) [24, 25, 26] to nanometric scales as large unilamellar vesicles (LUV) [27]. In the context of vaccine design, LUV offer substantial advantages over classical glycoarchitectures such as glycoclusters or multivalent glycosylated scaffolds [1, 2, 3, 4, 5, 6, 7, 8, 9, 10, 11, 12, 13, 14, 15, 16, 17, 18]. A key benefit lies in the synthetic accessibility and scalability of glycolipids, which can be obtained in substantial quantities—typically hundreds of milligrams—via conventional synthetic routes and purified efficiently using standard silica gel chromatography, thereby reducing both time and cost. Due to their amphiphilic nature and structural similarity to phospholipids, glycolipids spontaneously integrate into lipid bilayers and drive vesicle formation [28]. Why use engineered LUV? The resulting LUVs provide an exceptionally high capacity for glycan/epitopes display. To the best of our knowledge, in a few cases glycoclusters can typically accommodate up to 64 glycosyl moieties; in contrast, LUVs offer a dramatically larger presentation platform. For example, a liposome composed solely of 1‐palmitoyl‐2‐oleoyl‐*sn*‐glycero‐3‐phosphocholine (POPC) with a diameter of 100 nm has an estimated surface area of ~125,000 nm^2^ and can display up to ~190,000 entities per surface, according to measurements by Kučerka et al. [29] for POPC. This enables a vastly enhanced multivalent presentation of carbohydrate antigens. To the best of our knowledge, this represents one of the highest epitopes densities (in the nanometric scale) achievable in a modular and biocompatible format, positioning LUV as a powerful platform for next‐generation glycovaccine development. A notable example was recently reported by the research group of Chen and Zhang, who synthesized a “modified” cholesterol derivative that generates a Tn‐glycolipid antigen (Chol‐GalNAc/CpG), capable of simultaneously eliciting both humoral and cellular immune responses [19]. The vesicle size was characterized by transmission electron microscopy and found to be approximately 200 µm, which, in our assessment, may be excessively large for applications in human therapy. Importantly, the study did not address cytotoxicity assessments nor the absorption, distribution, metabolism, and excretion profile—critical parameters for evaluating the pharmacokinetics and safety of vaccine candidates. From a chemical standpoint, the carbonate linkage employed is known to lack enzymatic stability, potentially compromising in vivo durability. Furthermore, the use of TEC and simpler phospholipid‐based scaffolds compared to cholesterol ensures greater versatility in the synthesis of glycolipids. Additionally, employing Nanometer‐scale vesicles facilitates enhanced bioavailability by allowing LUVs to circulate through the bloodstream without posing a risk of vascular occlusion. In addition, in our long‐term studies, these constructs will demonstrate the ability to facilitate interactions with CD4^+^ T cells, leading to B‐cell activation and proliferation (unpublished). Moreover, engineered LUVs have the potential to increase the number of epitopes simultaneously recognized by B‐cell receptors and CD4^+^ T cells, thereby enhancing the overall efficacy of the vaccine. Notably, oncological vaccines based on this platform can elicit robust antitumor immunity by engaging B cells, CD4^+^ helper T cells, and CD8^+^ cytotoxic T cells [30, 31].

## 2 Results and Discussion

**FIGURE 1:**
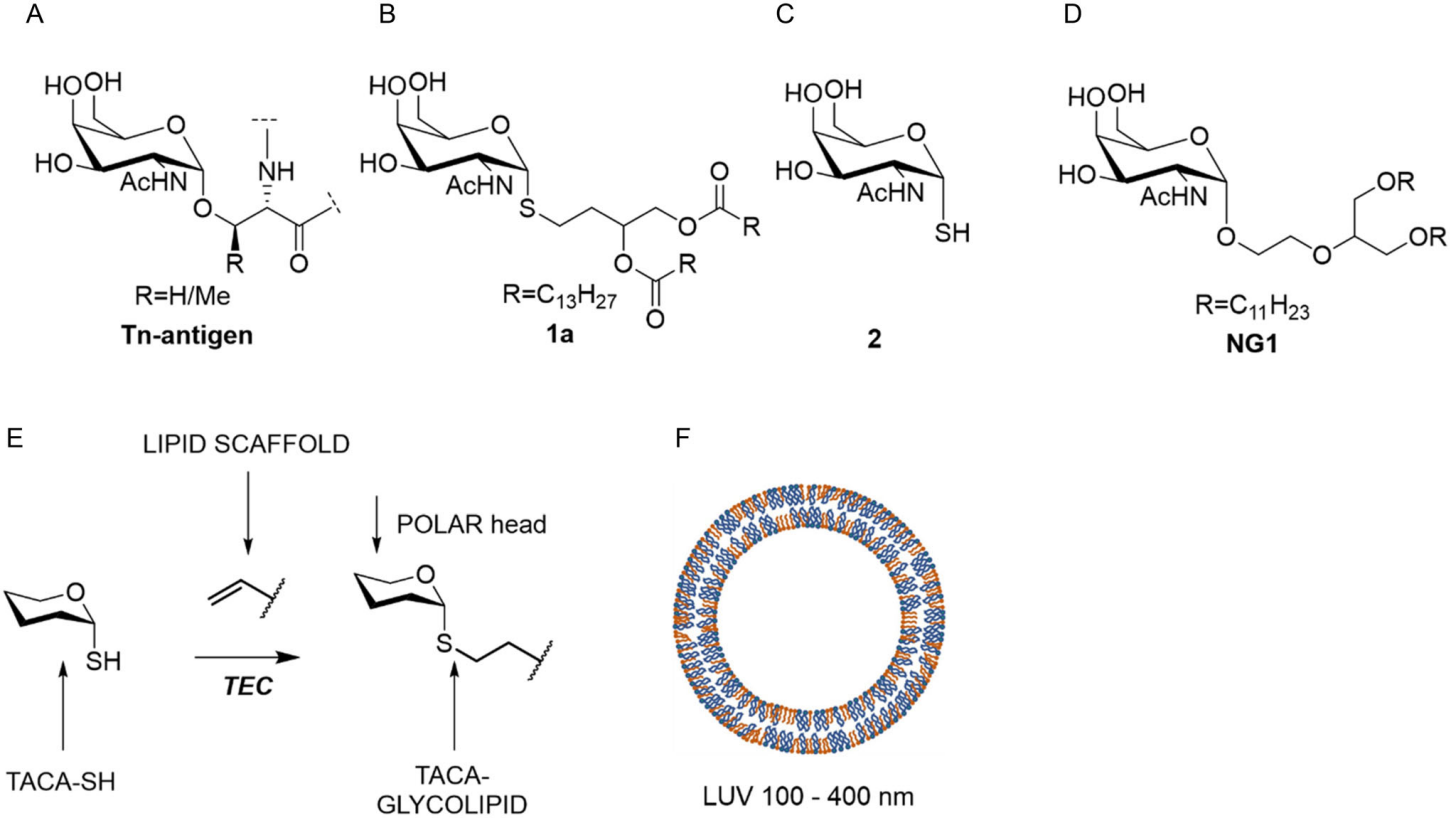
(A) The structure of the Tn Antigen; (B) the first glycolipid bearing a modified Tn antigen (**1a**) obtained by TEC [35]; (C) the structure of **2**; (D) structure of **NG1** [38]; (E) generic scheme for TEC applied to the production of thio‐glycolipids; (F) engineered LUVs; based on the surface occupied by the polar head of a molecule of POPC (68 Å^2^ = 0.68 nm^2^), each liposome of 100 nm diameter has a surface of ~125,000 nm^2^ constituted of ~190,000 molecules of POPC, the internal surface is not considered in this calculation.

As part of our rationale, we sought to investigate an often‐overlooked property of glyco‐clusters: their potential cytotoxicity. We decided to produce LUV‐bearing synthetic glycolipids using TEC, a methodology extensively optimized and routinely applied in our laboratory [12, 32, 33, 34]. These LUVs are called synthetic outer membrane vesicles (sOMV) [35] for their similarity with the outer membrane vesicles (OMVs) produced by Gram negative bacteria and extensively used in cancer vaccinology [36]. Mixtures of naturally occurring phospholipids and glycolipids bearing tumor‐associated carbohydrate antigens can be assembled into vesicles using well‐established methodologies. The resulting GUVs can be purified by simple vacuum filtration [37] and, upon application of standardized protocols, these GUVs can be downsized via extrusion to yield LUVs of defined diameters (Figure 1F) with a monodispersed population.

**SCHEME 1:**
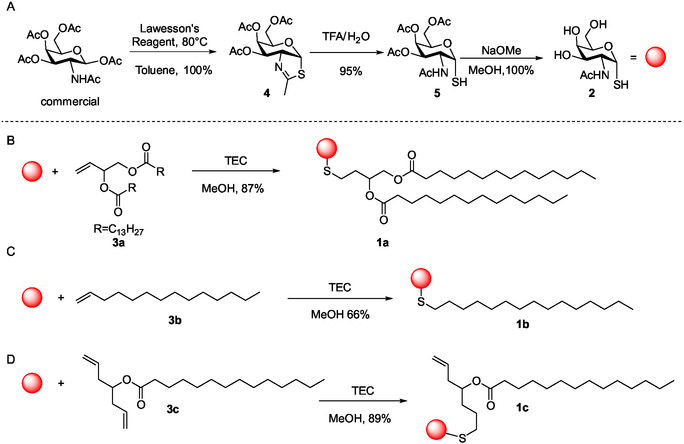
(A) Schematic preparation of mimic Tn‐SH antigen **2** [43]. (B–D) Synthesis of **1a** [35] and **1b**‐**1c** (this research), see Scheme S1 (Supporting Information) for further details.

To obtain biologically active glycolipids, we have selected the Thiol‐GalNAc as an analog of the Thomsen–Nouveau antigen (Tn). The Tn antigen is defined as a single N‐acetylgalactosamine (GalNAc) α‐linked to the hydroxyl group of a serine or a threonine residue (GalNAc‐*α*1‐*O*‐Ser/Thr; Figure 1A) and is a hallmark of aberrantly glycosylated mucins, frequently associated with malignant transformation and tumor progression [39]. For example the neoglycolipid **1a** is formed by coupling **2** to the lipophilic scaffold **3a** (Scheme 1B) through TEC. This stable molecule exhibited amphipathic properties as shown previously [35]. However, its toxicity remained unknown as well as its ability to form liposomes in the nanometric scale. We decided to carry out a new study preparing a small library of neoglycolipids, each formed by coupling different lipidic moieties (**3a**‐**3c**) to **2** via TEC but optimizing reaction conditions [23, 40, 41] and analyzing cytotoxicity and assessing their application in liposome preparation, both crucial properties before considering their use in nanomedicine as future vaccine components [42]. The synthesis of **2** (Scheme 1A) was carried out following the procedure described by Knapp and Myers [43] with a few modifications (under Ar atmosphere) that increased the overall yields up to 90%. This synthesis allows the preparation of solely the α anomer *via* acidic treatment of the thiazolidine intermediary **4**, followed by deacetylation in "Zemplén" conditions of **5** (Scheme 1A) [44]. Commercially available **3b** was used to prepare **1b** (Scheme 1C) and the product was purified by SiO_2_ flash chromatography using the isocratic eluent composed of CHCl_3_:MeOH:H_2_O (65:25:0.4 *v*/*v*/*v*, Eluent A) in 66% of yield. Compound **1c** was instead obtained from **3c** (Scheme 1D). Before irradiation, all the mixtures were saturated with argon at 1 atm for 30 min. Reactions were carried out in 5 mL test tubes under stirring and were irradiated with blue light (365 nm) at room temperature for 15 min and additional 15 min in the case of **1c**. TLC monitoring (DCM:MeOH 9:1, *v*/*v*) showed the complete consumption of the starting material **3a**, **3b,** or **3c**. The presence of disulfide was not observed. In the case of **3c** we have observed that only one thiol formed a stable thioether bond whereas one of the terminal akenes was unreacted. Two other sets of reactions have been performed: (i) **1c** bearing one thioether was submitted to a second reaction with 6 equivalents of **2 and** (ii) a second set of synthesis of **1d** was performed adding to **3c** up to 12 equivalents of **2**. In both cases a dramatical formation of the disulfide of **2** occurred. If formed, **1d** was probably present in traces and the yields of **1c** lowered to 25% per reaction. Thus, **1c** was reprepared to establish the yields that were around 50% of conversion using 3 or 6 eq of **2** per double bond in **3c** (see Scheme S1, Supporting Information).

**FIGURE 2:**
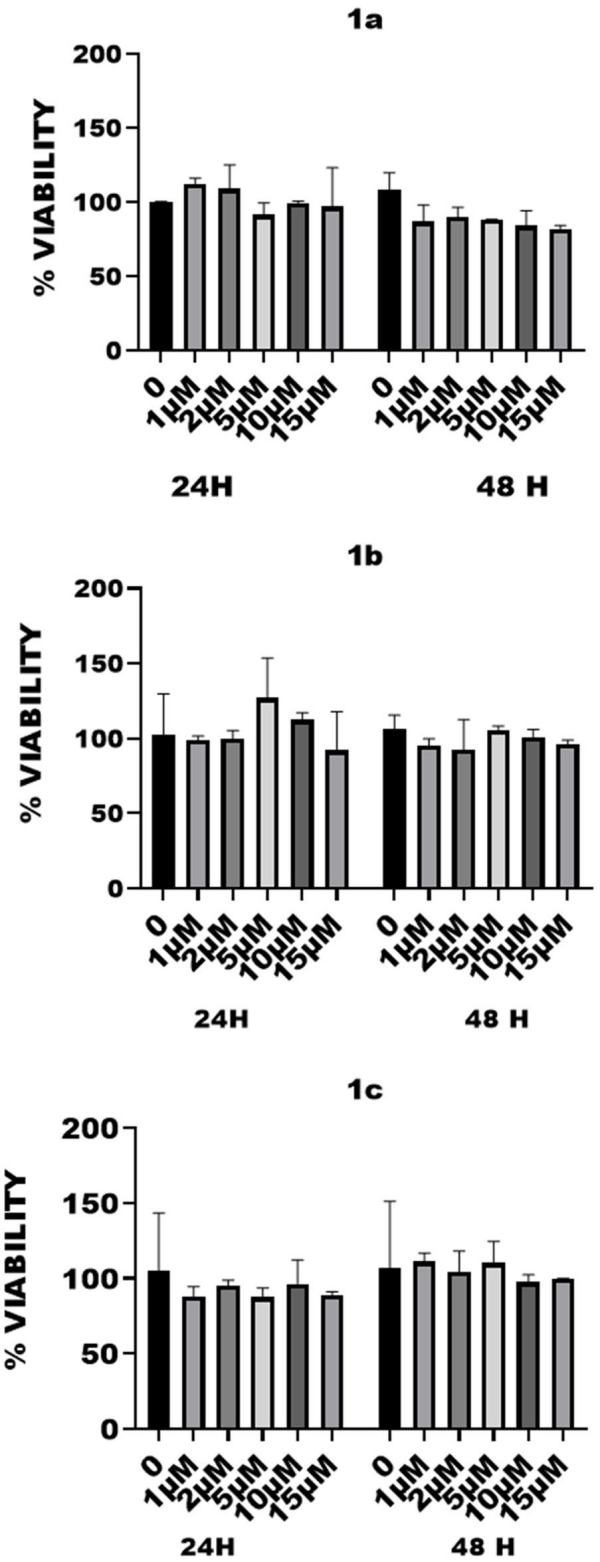
Results of the viability test on molecules **1a–1c** et **NG1** after 24 and 48 h of incubation and statistical analysis of the viability assays. Results were represented as mean ± standard error of the mean (SEM) and were expressed in percent as fold change compared to untreated control. For each analysis, three independent experiments were performed. To establish significance of our results, data were analyzed by the use of two‐sided Mann–Whitney *U* test. The level of significance was set at *p* < 0.05. Graphs and calculations were done using Prism and Instat 3, respectively (GraphPad software, California, USA). Statistical tests show that the results are not significant. The compounds have no significant effect on cell viability compared to the untreated control.

**FIGURE 3:**
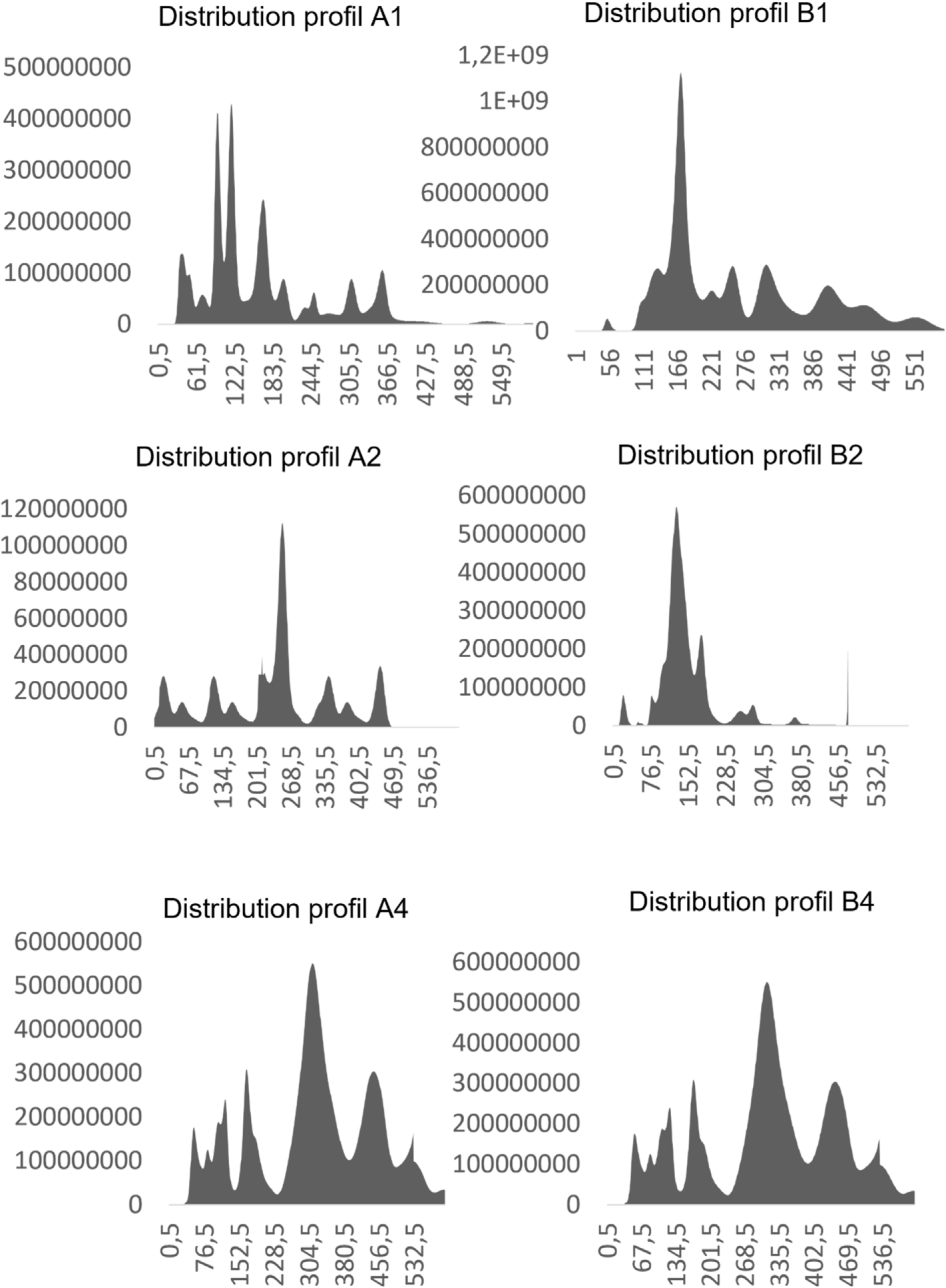
Size distribution of the population of s‐OMV (LUV) of nominal diameter 100 (A1), 200 (A2), and 400 (A4) nm containing **1a**, and size distribution of the population of s‐OMV (LUV) of nominal diameter 100 (B1), 200 (B2), and 400 (B4) nm containing **1b**.

All glycolipids exhibited no detectable toxicity after 24 and 48 h at concentrations up to 15 µM (Figure 2). We tested a range of compound concentrations on cells to assess toxicity. We went up to 15 µM because of limited compound solubility, which can cause precipitation at higher concentrations, and because of DMSO toxicity. DMSO becomes cytotoxic above approximately 0.5%–1% (v/v), depending on the cell line, which can confound the assay. Viability tests were conducted on murine vascular smooth muscle cell line as in previously reported researches [45, 46]. The absence of cytotoxicity (Table 1) has been a priority for us, a factor that is poorly reported in similar research [10, 19], while only the absence of immunogenicity of the scaffold is considered (i.e. ref. [47]). The absence of cytotoxicity was also detected for a sister molecule (**NG1**, Table 1, Figure S1, Supporting Information) presenting the GalNAc residue in the position *sn‐2* of glycerol moiety (Figure 1D). These compounds were used to prepare LUV with nominal diameter of 400, 200, and 100 nm (Table 2). The lipid composition was as follows: 49% of either **1a, 1b, 1c,** or **NG1**, 50% of POPC, and 1% of PA. The final concentration of each LUV was 0.01 mM. Only mixtures containing **1a** and **1b** hydrated enough to prepare LUV, whereas the lack of hydration for **1c** and **NG1** in mixtures can be explained for a too poor lipophilic part for the former, and the presence of the “polar head” in the *sn‐2* position for the latter (Scheme 1D and Figure 1D, respectively), that altered the conical shape required for the formation of a membrane bilayer or disrupted the membrane bilayer upon hydration [48]. The method used is a standardized one involving freeze–thaw cycling, followed by extrusion processes. The rapid freezing in liquid nitrogen (−78°C) and thawing at temperatures above the lipid phase transition (typically ~38°C) induces transient fusion and rupture events, facilitating internal reorganization of the vesicles. Following thermal cycling, the suspension is subjected to extrusion through polycarbonate membranes with defined pore sizes. This step disrupts residual multilamellar vesicles and selectively retains liposomes matching the filter diameter, yielding a population of LUVs with uniform size and improved reproducibility. A predominant and homogeneous size distribution was observed for all concentrations (A1‐A4 and B1‐BA) and liposome types (Table 2, 400–100 nm). Mixtures containing compound **1a** were labeled as series A, and those containing **1b** as series B. Homogeneous populations were present in each samples prepared using 100 nm (A1/B1), 200 nm (A2/B2), and 400 nm (A4/B4) pore sizes, as reported in Table 2. However, the size distribution does not align with the nominal membrane sizes but corresponds instead to the measured sizes—for example, A4 with a size distribution of 320 nm is comparable to B4 with 350 nm (see also Figure 3). The liposome concentrations (particles per mL) are also consistent across samples.

**TABLE 1:** Synthesis and biological evaluation of compounds **1a**‐**1c** and **NG1**.

Entry		Lipid scaffold	Product	Y%	liposomes (0.01 mM)	Cytotoxicity^c^
1	**2**	**3a**	**1a**	87	yes^a^	no
2	**2**	**3b**	**1b**	66	yes^a^	no
3	**2**	**3c** [23]	**1c**	70	no^b^	no
4			**NG1**	[38]	no^b^	no

a Only **1a** and **1b** in mixtures with POPC/PA molar ratio 49/50/1 and in a concentration of 0.01 M have successfully formed liposomes; average sizes are reported in Table 2.

b No hydration of the film produced by mixing **1c** or **NG1** with/POPC/PA 49/50/1 molar ratio 49/50/1 and in a concentration of 0.01 M.

c Compounds **1a**‐**1c** and **NG1** showed no toxicity at 15 µm after 24 and 48 h.

**TABLE 2:** NTA analysis for the liposomes of samples A (containing **1a**) and, B (containing **1b**).

Entry	**Mix** a **/Molecule**	**Size (nm)** b	Particle number (mL)	
1	A4/**1a**	320	9.23^10^ ± 4.18^9^	
2	A2/**1a**	240	1.01^11^ ± 1.12^10^	
3	A1/**1a**	118	2.93^10^ ± 3.39^9^	
4	B4/**1b**	350	9.23^10^ ± 4.18^9^	
5	B2/**1b**	172	1.01^11^ ± 1.12^10^	
6	B1/**1b**	130	3.31^10^ ± 2.47^9^	

a Means the mixtures containing **1a** or **1b**, PA and POPC (mol/ratio 49:1:50) for a final concentration 0.01 mM.

b The size distribution and concentration of the particles in the fraction were measured with NanoSight (NS300), see Experimental Section in Supporting Information for further information.

## 3 Conclusion

A set of glycolipids (**1a**–**1c**) was chemically and biologically evaluated. None of the compounds exhibited cytotoxicity. Among them, only **1a** and **1b** were used to generate nanosized sOMVs, with effective diameters ranging from 118 to 350 nm, which are either slightly above or below the nominal pore size of the membranes used for their extrusion from GUVs. The molar composition of each glycolipid in the vesicles was implemented up to 49%, in conformity with previously collected data from 2019 and 2023 [23, 35]. Synthetic procedures evidenced that phospholipid‐shaped glycolipids (like **1a**) prevalently formed using thiol‐ene reaction and produced vesicles upon hydration. Based on these findings, we are confident that liposome‐based vaccine platforms can be enhanced, particularly when antigens are integrated into the membrane bilayer. The data from this study, and those of others [10, 18, 19], support the feasibility of developing nanovaccines in the form of s‐OMVs. These promising results encourage us to further pursue the development of liposomes‐based vaccines incorporating CD4^+^ and CD8^+^ peptide antigens, adjuvants like PA, and potentially combinations of natural or fully synthetic phospholipid analogs [48]. In the next future, expected results are to trigger in a “one set” construct both humoral and cellular response, strengthening the immune system against the insurgence ofmalignancies.

## Supporting Information

Additional supporting information can be found online in the Supporting Information section. **Supporting** **Scheme S1:** Summary of the reactions carried out for the preparation of **1d**. **Supporting** **Fig.** **S1:** Results of the viability test on molecule **NG1** after 24 h of incubation. To establish significance of our results, data were analyzed by the use of two‐sided Mann‐Whitney U test. The level of significance was set at *p* < 0.05. Graphs and calculations were done using Prism and Instat 3, respectively (GraphPad software, California, USA). Statistical tests show that the results are not significant. The compounds have no significant effect on cell viability compared to the untreated control. **Supporting** **Fig.** **S2:** ^1^H NMR in DMSO‐d6 of compound **1b** (300 MHz). **Supporting** **Fig.** **S3:** ^13^C NMR in DMSO‐d6 of compound **1b** (75 MHz). **Supporting** **Fig.** **S4:** MS spectra of compound **1b**. **Supporting** **Fig.** **S5:** ^1^H NMR in DMSO‐d6 of compound **1c** (300 MHz). **Supporting** **Fig.** **S6:** ^13^C NMR in DMSO‐d6 of compound **1c** (75MHz). **Supporting** **Fig.** **S7:** HRMS spectra of compound **1c**. **Supporting** **Fig.** **S8** **–** **10:** ^1^H, g‐COSY, ^13^C and HSQC of compound **1c** in MeOD.

## Conflicts of Interest

The authors declare no conflicts of interest.

## Supporting information

Supplementary Material

## Data Availability

The data that support the findings of this study are available on request from the corresponding author.

## References

[1] S. E. Sherman , Q. Xiao , and V. Percec , “Mimicking Complex Biological Membranes and Their Programmable Glycan Ligands with Dendrimersomes and Glycodendrimersomes,” Chemical Reviews 117 (2017): 6538–6631, 10.1021/acs.chemrev.7b00097. 28417638

[2] E. C. Wrobel , L. S. de Lara , Â. de Fátima , and O. N. Oliveira , “Nanoarchitectonics and Simulation on the Molecular‐Level Interactions between, *p‐*, Sulfonic Acid Calix[4]arene and *Langmuir* Monolayers Representing Healthy and Cancerous Cell Membranes,” Langmuir 40 (2024): 27010–27027. 39663612 10.1021/acs.langmuir.4c03948 PMC11673576

[3] X. Zhang , H. Cong , B. Yu , and Q. Chen , “Recent Advances of Water‐Soluble Fullerene Derivatives in Biomedical Applications,” Mini‐Reviews in Organic Chemistry 16 (2018): 92–99.

[4] N. Bognanni , M. Viale , A. Distefano , et al., “Cyclodextrin Polymers as Delivery Systems for Targeted Anti‐Cancer Chemotherapy,” Molecules 26 (2021): 6046. 34641590 10.3390/molecules26196046 PMC8512365

[5] S. D. Kuduk , J. B. Schwarz , X. T. Chen , et al., “Synthetic and Immunological Studies on Clustered Modes of Mucin‐Related Tn and TF O‐Linked Antigens: The Preparation of a Glycopeptide‐Based Vaccine for Clinical Trials against Prostate Cancer,” Journal of the American Chemical Society 120 (1998): 12474–12485.

[6] S. J. Danishefsky and J. R. Allen , “From the Laboratory to the Clinic: A Retrospective on Fully Synthetic Carbohydrate‐Based Anticancer Vaccines,” Angewandte Chemie International Edition 39 (2000): 836–863. 10760879 10.1002/(sici)1521-3773(20000303)39:5<836::aid-anie836>3.0.co;2-i

[7] S. J. Keding , A. Endo , and S. J. Danishefsky , “Synthesis of Non‐Natural Glycosylamino Acids Containing Tumor‐Associated Carbohydrate Antigens,” Tetrahedron 59 (2003): 7023–7031.

[8] H. Herzner and H. Kunz , “Spacer‐Separated Sialyl LewisX Cyclopeptide Conjugates as Potential E‐Selectin Ligands,” Carbohydrate Research 342 (2007): 541–557. 17027941 10.1016/j.carres.2006.09.012

[9] S. Dziadek , A. Hobel , E. Schmitt , and H. Kunz , “A Fully Synthetic Vaccine Consisting of a Tumor‐Associated Glycopeptide Antigen and a T‐Cell Epitope for the Induction of a Highly Specific Humoral Immune Response,” Angewandte Chemie International Edition 44 (2005): 7630–7635. 16247815 10.1002/anie.200501594

[10] C. Pifferi , B. Thomas , D. Goyard , N. Berthet , and O. Renaudet , “Heterovalent Glycodendrimers as Epitope Carriers for Antitumor Synthetic Vaccines,” Chemistry ‐ A European Journal 23 (2017): 16283–16296. 28845889 10.1002/chem.201702708 PMC6175327

[11] M. Fiore , B. Thomas , V. Duléry , P. Dumy , and O. Renaudet , “Synthesis of Multi‐Antigenic Platforms as Vaccine Candidates against Cancers,” New Journal of Chemistry 37 (2013): 286–289.

[12] S. Fallarini , A. Brittoli , M. Fiore , et al., “Immunological Characterization of a Rigid *α*‐Tn Mimetic on Murine iNKT and Human NK Cells,” Glycoconjugate Journal 34 (2017): 553–562. 28573337 10.1007/s10719-017-9775-6

[13] B. Richichi , B. Thomas , M. Fiore , et al., “A Cancer Therapeutic Vaccine Based on Clustered Tn‐Antigen Mimetics Induces Strong Antibody‐Mediated Protective Immunity,” Angewandte Chemie International Edition 53 (2014): 11917–11920. 25168881 10.1002/anie.201406897 PMC4564297

[14] B. Liet , E. Laigre , D. Goyard , et al., “Multifunctional Glycoconjugates for Recruiting Natural Antibodies against Cancer Cells,” Chemistry – A European Journal 25 (2019): 15508–15515. 31613028 10.1002/chem.201903327 PMC6916168

[15] E. Richard , C. Pifferi , M. Fiore , et al., “Chemobacterial Synthesis of a Sialyl‐Tn Cyclopeptide Vaccine Candidate,” ChemBioChem 18 (2017): 1730–1734. 28632300 10.1002/cbic.201700240

[16] B. Richichi , G. Comito , O. Renaudet , et al., “Role of a Preorganized Scaffold Presenting Four Residues of a GM‐3 Lactone Mimetic on Melanoma Progression,” ACS Medicinal Chemistry Letters 7 (2016): 28–33. 26819661 10.1021/acsmedchemlett.5b00283 PMC4716596

[17] I. Bettahi , G. Dasgupta , O. Renaudet , et al., “Antitumor Activity of a Self‐Adjuvanting Glyco‐Lipopeptide Vaccine Bearing B Cell, CD4+ and CD8+ T Cell Epitopes,” Cancer Immunol, Immunother 58 (2009): 187–200. 18584174 10.1007/s00262-008-0537-y PMC11030914

[18] C. Pifferi , A. Ruiz‐de‐Angulo , D. Goyard , et al., “Chemical Synthesis and Immunological Evaluation of New Generation Multivalent Anticancer Vaccines Based on a Tn Antigen Analogue,” Chemical Science 11 (2020): 4488–4498. 34122907 10.1039/d0sc00544d PMC8159477

[19] L. Yao , L. Wu , R. Wang , et al., “Liposome‐Based Carbohydrate Vaccine for Simultaneously Eliciting Humoral and Cellular Antitumor Immunity,” ACS Macro Letters 11 (2022): 975–981. 35833848 10.1021/acsmacrolett.2c00291

[20] R. E. Bird , S. A. Lemmel , X. Yu , and Q. A. Zhou , “Bioorthogonal Chemistry and Its Applications,” Bioconjugate Chemistry 32 (2021): 2457–2479. 34846126 10.1021/acs.bioconjchem.1c00461

[21] S. Cecioni , J. Praly , S. E. Matthews , M. Wimmerová , A. Imberty , and S. Vidal , “Rational Design and Synthesis of Optimized Glycoclusters for Multivalent Lectin–Carbohydrate Interactions: Influence of the Linker Arm,” Chemistry – A European Journal 18 (2012): 6250–6263. 22488581 10.1002/chem.201200010

[22] F. Peri , “Clustered Carbohydrates in Synthetic Vaccines,” Chemical Society Reviews 42 (2013): 4543–4556. 23250562 10.1039/c2cs35422e PMC3654047

[23] D. Fayolle , N. Berthet , B. Doumeche , O. Renaudet , P. Strazewski , and M. Fiore , “Towards the Preparation of Synthetic Outer Membrane Vesicle Models with Micromolar Affinity to Wheat Germ Agglutinin Using a Dialkyl Thioglycoside,” Beilstein Journal of Organic Chemistry 15 (2019): 937–946. 31164930 10.3762/bjoc.15.90 PMC6541351

[24] P. Dimova , R. Stano , P. Marques , and C. M. Walde , “Preparation Methods for Giant Unilamellar Vesicles,” in The Giant Vesicle Book (Taylor & Francis Group, Boca Raton, 2020).

[25] P. Walde , K. Cosentino , H. Engel , and P. Stano , “Giant Vesicles: Preparations and Applications,” ChemBioChem 11 (2010): 848–865. 20336703 10.1002/cbic.201000010

[26] J. T. Kindt , J. W. Szostak , and A. Wang , “Bulk Self‐Assembly of Giant, Unilamellar Vesicles,” ACS Nano 14 (2020): 14627–14634. 32602696 10.1021/acsnano.0c03125 PMC8172239

[27] M. J. Hope , M. B. Bally , G. Webb , and P. R. Cullis , “Production of Large Unilamellar Vesicles by a Rapid Extrusion Procedure. Characterization of Size Distribution, Trapped Volume and Ability to Maintain a Membrane Potential,” Biochimica et Biophysica Acta (BBA) ‐ Biomembranes 812 (1985): 55–65. 23008845 10.1016/0005-2736(85)90521-8

[28] W. Curatolo , “The Physical Properties of Glycolipids,” Biochimica et Biophysica Acta (BBA) ‐ Reviews on Biomembranes 906 (1987): 111–136. 3297151 10.1016/0304-4157(87)90008-6

[29] N. Kučerka , S. Tristram‐Nagle , and J. F. Nagle , “Structure of Fully Hydrated Fluid Phase Lipid Bilayers with Monounsaturated Chains,” The Journal of Membrane Biology 208 (2006): 193–202. 10.1007/s00232-005-7006-8 16604469

[30] L. Cipolla , F. Peri , and C. Airoldi , “Glycoconjugates in Cancer Therapy,” Anti‐Cancer Agents in Medicinal Chemistry 8 (2008): 92–121. 18220509 10.2174/187152008783330815

[31] Z. Gan and R. Roy , “Sialoside Clusters as Potential Ligands for Siglecs (sialoadhesins),” Canadian Journal of Chemistry 80 (2002): 908–916.

[32] D. Goyard , V. Baldoneschi , A. Varrot , et al., “Multivalent Glycomimetics with Affinity and Selectivity toward Fucose‐Binding Receptors from Emerging Pathogens,” Bioconjugate Chemistry 29 (2018): 83–88. 29240403 10.1021/acs.bioconjchem.7b00616

[33] M. Fiore , N. Berthet , O. Renaudet , and V. Barbier , “New Glycopolymers as Multivalent Systems for Lectin Recognition,” Medchemcomm 5 (2014): 1202–1207.

[34] M. Fiore , G. C. Daskhan , B. Thomas , and O. Renaudet , “Orthogonal Dual Thiol‐Chloroacetyl and Thiol‐ene Couplings for the Sequential One‐Pot Assembly of Heteroglycoclusters,” Beilstein Journal of Organic Chemistry 10 (2014): 1557–1563. 25161711 10.3762/bjoc.10.160 PMC4142873

[35] C. Chieffo , A. Comte , P. Strazewski , and M. Fiore , “Synthetic Outer Membrane Vesicles Bearing Tn Antigen,” European Journal of Organic Chemistry 26 (2023), 10.1002/ejoc.202300820.

[36] M.‐Y. Chen , T.‐W. Cheng , Y.‐C. Pan , et al., “Endotoxin‐Free Outer Membrane Vesicles for Safe and Modular Anticancer Immunotherapy,” ACS Synthetic Biology 14 (2025): 148–160. 39763210 10.1021/acssynbio.4c00483 PMC11744915

[37] D. Fayolle , M. Fiore , P. Stano , and P. Strazewski , “Rapid Purification of Giant Lipid Vesicles by Microfiltration,” PLoS One 13 (2018): e0192975. 29451909 10.1371/journal.pone.0192975 PMC5815610

[38] N. Laurent , D. Lafont , and P. Boullanger , “Syntheses of *α*‐d‐Galactosamine Neoglycolipids,” Carbohydrate Research 341 (2006): 823–835. 16516175 10.1016/j.carres.2006.02.013

[39] C. Chen , A. Patel , L. Demirkhanyan , and C. S. Gondi , “The Role of Mucins in Cancer and Cancer Progression: A Comprehensive Review,” Current Issues in Molecular Biology 47 (2025): 406. 40699805 10.3390/cimb47060406 PMC12191488

[40] M. Fiore , A. Marra , and A. Dondoni , “Photoinduced Thiol−Ene Coupling as a Click Ligation Tool for Thiodisaccharide Synthesis,” The Journal of Organic Chemistry 74 (2009): 4422–4425. 19422247 10.1021/jo900514w

[41] M. Fiore , A. Chambery , A. Marra , and A. Dondoni , “Single and Dual Glycoside Clustering around Calix[4]arene Scaffolds via Click Thiol‐ene Coupling and Azide‐Alkyne Cycloaddition,” Organic & Biomolecular Chemistry 7 (2009): 3910–3913. 19763289 10.1039/b912686d

[42] J. Zhai , C. Fong , N. Tran , and C. J. Drummond , “Non‐Lamellar Lyotropic Liquid Crystalline Lipid Nanoparticles for the Next Generation of Nanomedicine,” ACS Nano 13 (2019): 6178–6206. 31082192 10.1021/acsnano.8b07961

[43] S. Knapp and D. S. Myers , “Synthesis of *α*‐GalNAc Thioconjugates from an *α*‐GalNAc Mercaptan,” The Journal of Organic Chemistry 67 (2002): 2995–2999. 11975558 10.1021/jo0110909

[44] B. Ren , M. Wang , J. Liu , J. Ge , X. Zhang , and H. Dong , “Zemplén Transesterification: A Name Reaction that Has Misled Us for 90 Years,” Green Chemistry 17 (2015): 1390–1394.

[45] C. Chieffo , E. Altamura , G. Pilet , S. Mebarek , P. Strazewski , and M. Fiore , “Microwave‐Assisted Syntheses of Rhodamine, Rhodol, and Fluorescein Derivatives,” ChemPlusChem 88 (2023): e202300189, 10.1002/cplu.202300189. 37442786

[46] C. Chieffo , E. Altamura , L. Ben Trad , et al., “Comprehensive Characterization of an Off/On Rhodol‐Based Lysosomal Tracker for Orthogonal Cellular Analysis by Confocal Imaging,” ChemBioChem 24 (2023): e202200513, 10.1002/cbic.202200513. 36420688

[47] O. Renaudet and P. Dumy , “Chemoselectively Template‐Assembled Glycoconjugates as Mimics for Multivalent Presentation of Carbohydrates,” Organic Letters 5 (2003): 243–246. 12556162 10.1021/ol0270935

[48] P. Albanese , C. Amante , S. Cataldini , et al., “1‐(4‐Aminophenoxy)‐3‐(alkyl)propane‐2‐Ols as Building Blocks for the Preparation of Membranogenic Compounds,” ChemistrySelect 10 : 2025): e03367, 10.1002/slct.202503367.

