# Supplementary material for: Liposome‐Based Potential Vaccines Platforms that Are Noncytotoxic
Source: ChemistryOpen. 2026 Jan 11;15(1):e202500530. doi: 10.1002/open.202500530 (PMC12791030; doi:10.1002/open.202500530)
Supplement: Supplementary file 1 — Supplementary Material [file OPEN-15-e202500530-s001.pdf]

Matherials and Methods

All the reagents and solvents were purchased from Sigma-Aldrich, TCI Europe, Fischer Scientific, BLD pharma and were used without further purification. Solvents were purchased from Thermo-Fischer Scientific (LC/MS grade). The water used was of Milli-Q grade, 18.2 MΩ·cm at 25°C with 3 ppb TOC, purified using an ELGA Purelab option-Q (Wasquehal, France). Thin-layer chromatography (TLC) was carried out on aluminium sheets coated with silica gel 60 F254 (Merck, Kenilworth, USA). TLC plates were inspected by UV light (λ = 254 nm) or developed by treatment with a solution of 10% H_2SO_4 in EtOH and heat.

Electrospray-ionization mass spectra (ESI-MS) were recorded using a Q-Tof Micromass spectrometer. NMR spectra were recorded in DMSO-d_6, D_2O, $CDCl_3$ or MeOD-d_4 on a Bruker Avance 300MHz spectrometer at 300, and 75 MHz and on a Bruker Avance 400 MHz at 400 and 100 MHz for ^{1}H, and ^{13}C, respectively. Chemical shifts of solvents (DMSO-d6: d_H=2.50 and d_C=39.51; $CDCl_3$: d_H=7.26 and d_C=77.23; MeOD: d_H=3.31 and d_C=49.15) served as internal references. Signal shapes and multiplicities are abbreviated as br (broad), s (singlet), d (doublet), t (triplet), q (quartet) and m (multiplet). Where possible, a scalar coupling constant *J* is given in Hertz (Hz).

The size distribution and concentration of the particles in the fraction were measured with NanoSight (NS300) (Malvern Panalytical Ltd., Malvern, UK) equipped with nanoparticle tracking analysis (NTA) software (NanoSight NS300; Malvern Panalytical Ltd., Malvern, UK). All purified samples were diluted in PBS buffer (1 :1000) to a volume of 1mL at an ideal concentration of 20–50 particles per frame and injected into the machine's specimen chamber with a 1-mL sterile syringe. Measurements of the buffer's pH were systematically performed. For each measurement, five acquisitions of 1 min were recorded at 25◦C.

Compound **1a** was prepared accordingly with reference using MeOH as solvent instead of DMF and this procedure was applied for the preparation of **1b** and **1c** performed in TEC as well.

Compound **1b**: In a 5 ml test tube, a stirred solution of **2** (56 mg, 0.24 mmol, 3 eq.), **3b** (15mg, 0.08 mmol, 1 eq.) and DPAP (5 mg, 0.02 mmol, 0.02 eq) in 2000 µL of MeOH was irradiated (365 nm) at room temperature for 60 min under saturated argon atmosphere. TLC (DCM: MeOH 9:1, *v*/*v*) showed the complete consumption of the starting material **3b**. The reaction mixture was concentrated in vacuo and the crude mixture was purified by silica flash chromatography (Eluent A) giving 23 mg of **1b** (Yield: 66%) ^{1}H NMR (400 MHz, MeOD) δ_H 4.46 (d, *J* = 12.0 Hz, 1H), 3.86 (dd, *J* = 12.0 Hz, 1H), 3.72 (t, *J* = 12.0 Hz, 1H), 3.70–3.64 (m, 2H), 3.32 (t, *J* = 12.0 Hz, 1H), 3.22 (t, *J* = 12.0 Hz, 1H, partially masked by HOD signal), 2.70 (m, 2H) 1.97 (s, 3H), 1.61 (m, 2H), 1.29 (s, 14H), 0.90 (t, *J* = 8.0 Hz, 3H); ^{13}C NMR (100.0 MHz, MeOD) δ_C 12.9, 21.6, 22.3, 25.6, 28.6, 28.9, 29.1, 29.2, 29.3–29.4, 54.9, 61.4, 70.6, 76.0, 80.8, 84.3; *m/z*: found 434.4 as [M+H]; calculated 433.29

Compound **1c.** In a 5 ml test tube, a stirred solution of **2** (206 mg, 0.6 mmol, 6 eq.), **3c** (34.5 mg, 0.1 mmol, 1 eq.) and DPAP (7.68 mg, 0.03 mmol, 0.03 eq) in 2000 µL of MeOH was irradiated (365 nm) at room temperature for 120 min under saturated argon atmosphere. TLC (DCM: MeOH 9:1, *v*/*v*) showed the complete consumption of the starting material **3c**. The reaction mixture was concentrated in vacuo and the crude mixture was purified by silica flash chromatography (Eluent A) giving 50 mg of **1c** (Yield: 89%) ^{1}H NMR (300 MHz, DMSO-d_6) δ_H 5.34 (m, 4H), 5.08 (m, 1H), 4.34–4.21 (m, 2H), 3.71 (d, *J* = 4.0 Hz, 2H), 2.35–2.21 (m, 4H), 2.00 (d, *J* = 8.0 Hz, 4H), 1.61 (d, *J* = 4.0 Hz, 4H), 1.27 (d, *J* = 16.1 Hz, 34H), 0.87 (t, *J* = 8.0 Hz, 6H); ^{13}C NMR (75.0 MHz, DMSO-d_6) δc 22.7, 24.8, 24.9, 25.6, 27.0, 27.1, 27.2, 29.0–29.2, 29.3, 29.4, 29.7, 29.8, 31.9, 34.0, 34.3, 61.5, 62.0, 66.0, 72.1, 129.7, 130.0, 173.4, 173.8, 173.9; *m/z*: found 560.5 as [M+H]; calculated 559.25

Compound **1d**. i) In a 5 ml test tube 40 mg of **1c** (0.07 mmol), **2** (412 mg, 0.12 mmol, 12 eq) and (15 mg of DPAP, 0.06 mmol) were solubilized in MeOH (2000µL) and stirred under argon for 30 minutes until the solution became clear. The solution was irradiated at 365 nm for 1, 2, 4, 8 and 11h under atmosphere of Ar. Only the formation of disulfide was observed and if present traces of **1d** were present (MS spectra messy); ii) In a 5 ml test tube, a stirred solution of **2** (412 mg, 0.12 mmol, 12 eq) and (15 mg of DPAP, 0.06 mmol) were solubilized in MeOH (2000µL) TLC (DCM: MeOH 9:1, *v*/*v*) showed the complete consumption of the starting material **3c**. The reaction mixture was concentrated in vacuo and the crude mixture was purified by silica flash chromatography (Eluent A) giving 8 mg of **1c** (Yield: <25%)

Preparation of LUV: **Preparation of LUV**: The giant unilamellar vesicles (GUV) obtained through freeze–thaw cycles were extruded through polycarbonate membranes with varying pore sizes (400 nm, 200 nm, and 100 nm; Whatman, Nucleopore). The resulting nanosized s-OMV were characterized by nanoparticle tracking analysis (NTA, see materials and method section). A thin lipid film was prepared by dissolving the lipid mixtures in a 9:1 $CHCl_3$: MeOH solution in a 5 mL round-bottom flask as reported.

The organic solvent was carefully evaporated, and the residual lipid film was dried overnight under vacuum (13 mmHg). The total lipid concentration in the final mixtures was 0.01 mM per mL of HEPES buffer (20 mM, pH 8.0). The lipid films were rapidly hydrated with 1 mL of buffer and subjected to vigorous vortexing at room temperature for 3 minutes. The resulting mixtures underwent six freeze–thaw cycles (from –78 °C to 30 °C). This process minimized the formation of multilamellar giant vesicles (MGV), promoting instead the formation of multilamellar large vesicles (MLV) prior to extrusion. Mixtures containing compound **1c** showed no vesicle formation, even after incubation at 37 °C for 24 hours, and were excluded from further analysis.

Biological methods for cytotoxicity. Murine Vascular Smooth Muscle Cell line MOVAS was obtained from ATCC (American Type Culture Collection). MOVAS cells were plated at a density of 25,000 cells/cm2 in 12-well plates (Corning Inc, Boulogne-Billancourt, FR) in so-called growth medium consisting of Dulbecco's modified Eagle's medium (DMEM) containing 10% (*v*:*v*) fetal bovine serum (FBS), 100 U. mL-1 penicillin and 100 µg. mL-1 streptomycin (both from Sigma Aldrich, Lyon, FR). Cultures were maintained in a humidified atmosphere consisting of 95% air and 5% CO_2 at 37 °C. For the MTT test, (3- (4,5-dimethylthiazol-2-yl)-2,5-diphenyltetrazolium bromide) was used, cells were plated in 12-well plates in growth medium supplemented with different compounds at different concentrations.

Cell viability assay. The viability of cultured cells (MOVAS cell line) was measured using the MTT colorimetric assay (Roche Diagnostics, Meylan, France) as described previously. Then the MTT labeling reagent (0.5 mg mL-1 final concentration) was added to each well. The cells were further incubated for 4h. 100 µl of solubilization solution (10% SDS (g:mL) in 0.01M HCl) were then added and plates were allowed to stand overnight at 37°C in a humidified atmosphere. Cell viability was directly related to the difference in absorbance measured at 550 and 690 nm using a Tecan Infinite M200 (Salzburg, Austria) micro-titre plate reader. Results were normalized relative to their respective controls taken as 100. For each probe, three distinct sample pools were analyzed in a triplicate manner (n = 9).

Statistical analysis

Results were represented as mean ± standard error of the mean (SEM) and were expressed in % as fold change compared to untreated control. For each analysis, 3 independent experiments were performed. To establish significance of our results, data were analyzed by the use of two-sided Mann-Whitney U test. The level of significance was set at $p < 0.05$. Graphs and calculations were done using Prism and Instat 3, respectively (GraphPad software, California, USA). Statistical tests show that the results are not significant. The compounds have no significant effect on cell viability compared to the untreated control.

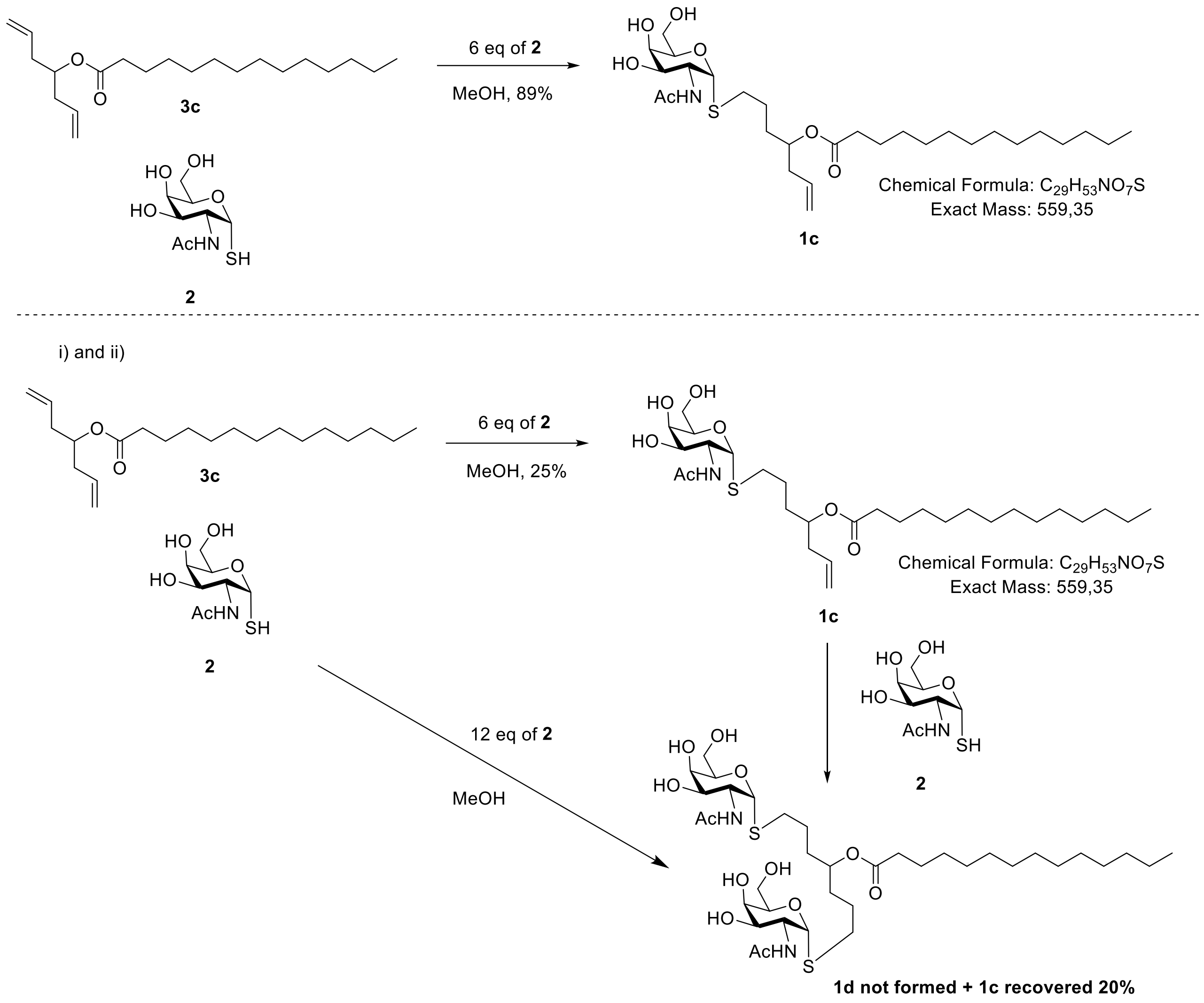

Scheme S1. Summary of the reactions carried out for the preparation of **1d**

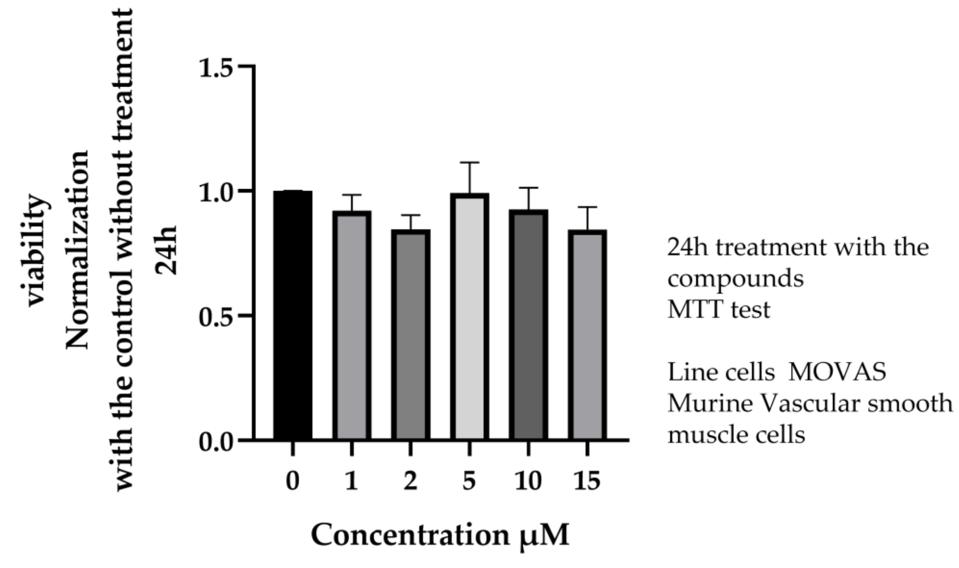

Figure S1. Results of the viability test on molecule **NG1** after 24 h of incubation. To establish significance of our results, data were analyzed by the use of two-sided Mann-Whitney *U* test. The level of significance was set at $p < 0.05$. Graphs and calculations

were done using Prism and Instat 3, respectively (GraphPad software, California, USA). Statistical tests show that the results are not significant. The compounds have no significant effect on cell viability compared to the untreated control

Appendix A

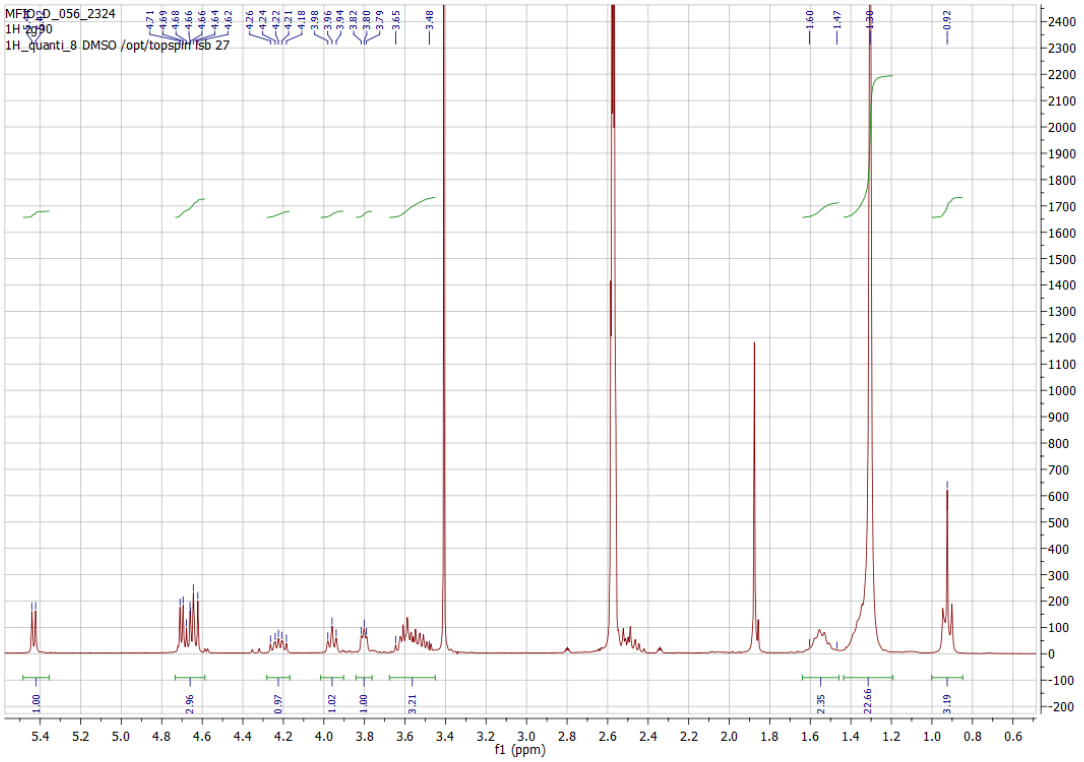

Figure S2 ^{1}H NMR in DMSO-d6 of compound **1b** (300MHz)

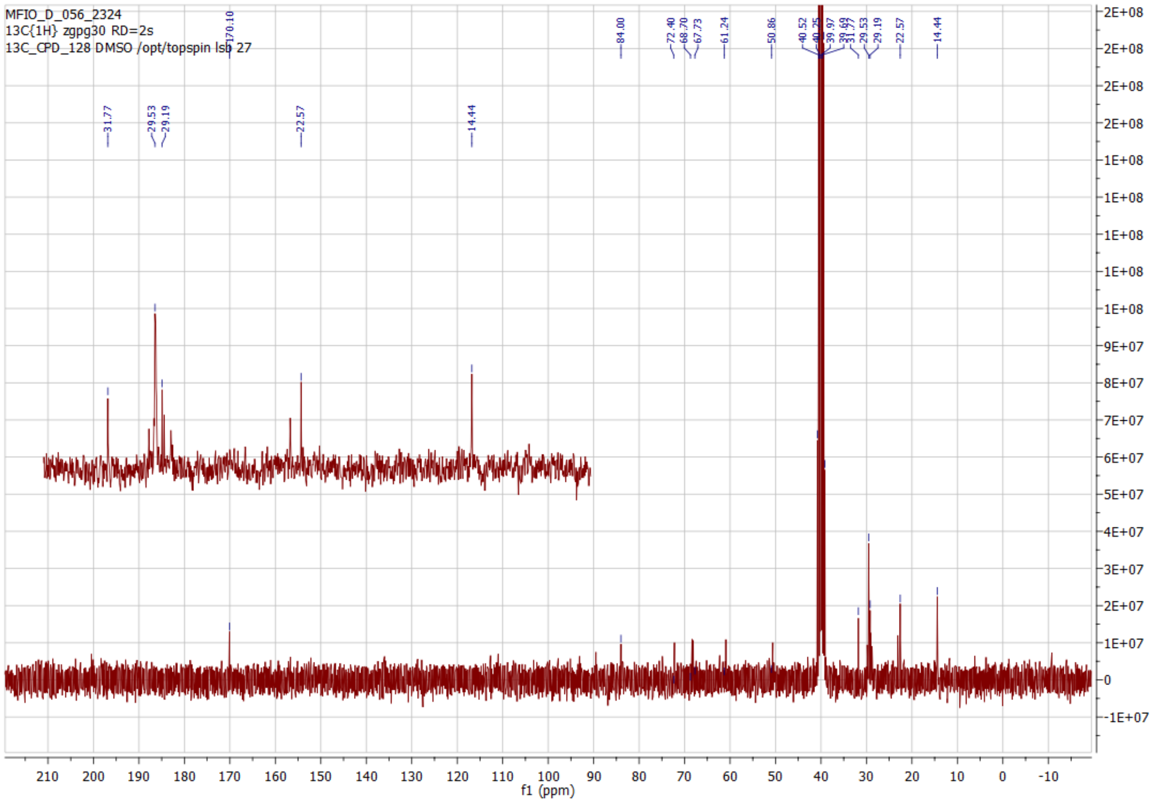

Figure S3 ^{13}C NMR in DMSO-d_6 of compound **1b** (75 MHz)

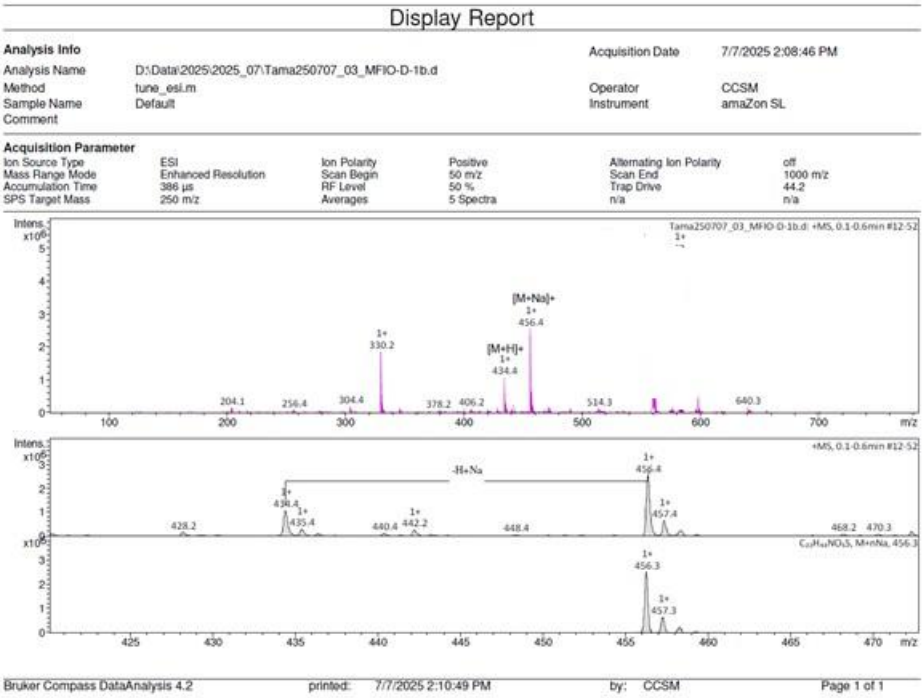

Figure S4 MS spectra of compound **1b**

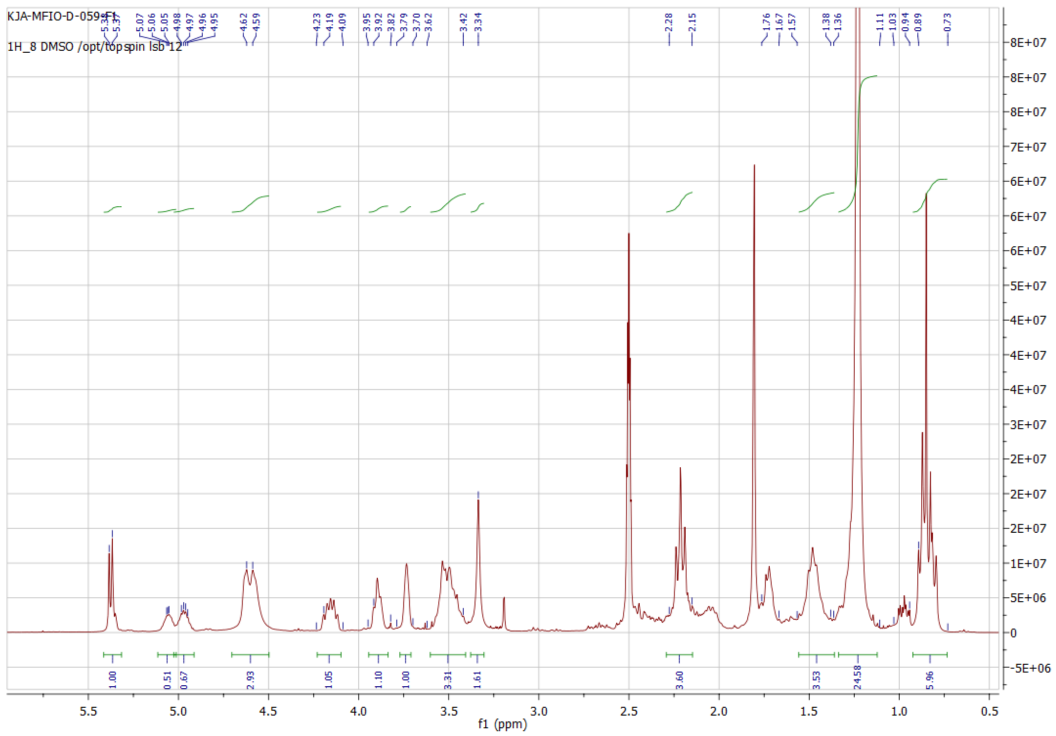

Figure S5 ^{1}H NMR in DMSO-d_6 of compound **1c** (300 MHz)

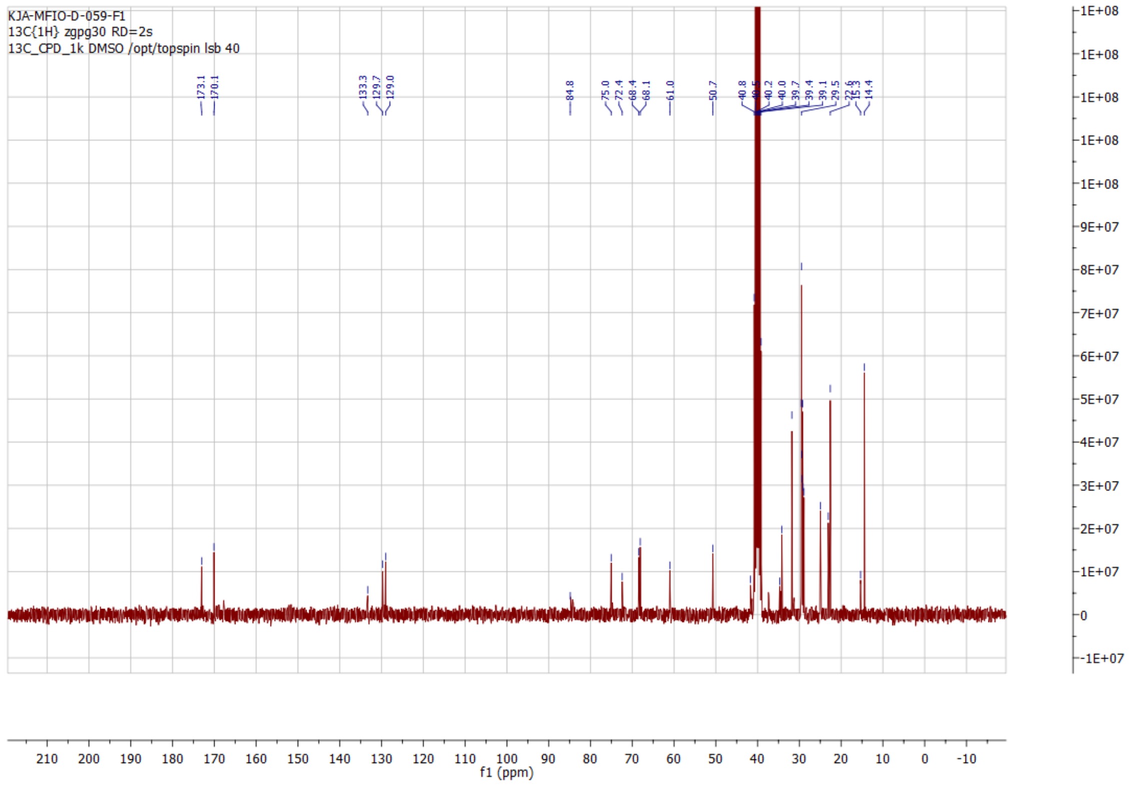

Figure S6. ^{13}C NMR in DMSO-d_6 of compound **1c** (75MHz)

CENTRE COMMUN DE SPECTROMETRIE DE MASSE

Analysis Info

Analysis Name	Impact2LC_250905_02_MFIO-D-059-1c.d		
Method	Tune_pos_StandardAV.m	Acquisition Date	9/5/2025 3:00:35 PM
Comment		Instrument / Ser#	impact II 1825265.1 0099

Acquisition Parameter

Source Type	ESI	Ion Polarity	Positive	Set Nebulizer	0.3 Bar
Focus	Active	Set Capillary	4500 V	Set Dry Heater	200 °C
Scan Begin	50 m/z	Set End Plate Offset	-500 V	Set Dry Gas	4.0 l/min
Scan End	1000 m/z	Set Collision Cell RF	750.0 Vpp	Set Divert Valve	Source

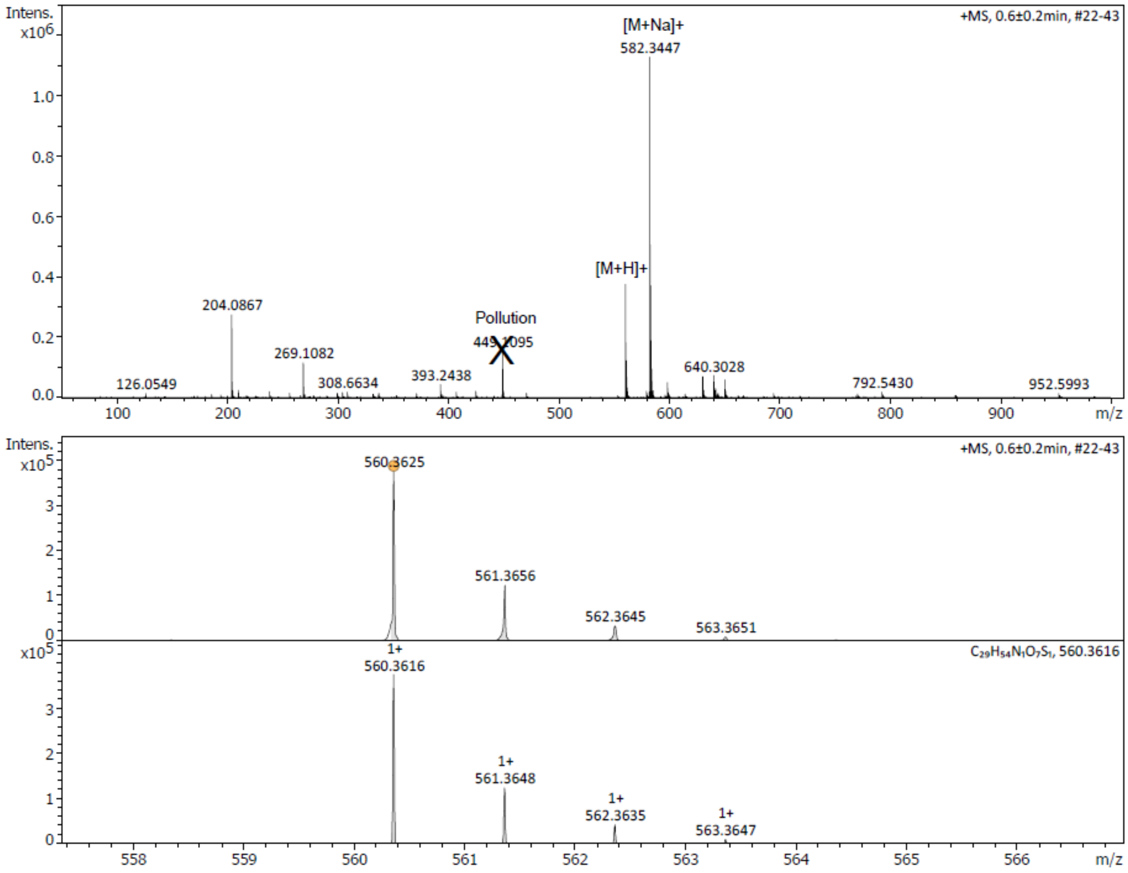

Meas. m/z	Ion Formula	m/z	Sum Formula	err [ppm]	mSigma	Adduct	z
560.3625	C29H54NO7S	560.3616	C29H53NO7S	-1.6	13.6	M+H	1+
582.3447	C29H53NNaO7S	582.3435	C29H53NO7S	-2.1	15.1	M+Na	1+

Figure S7 HRMS spectra of compound **1c**;

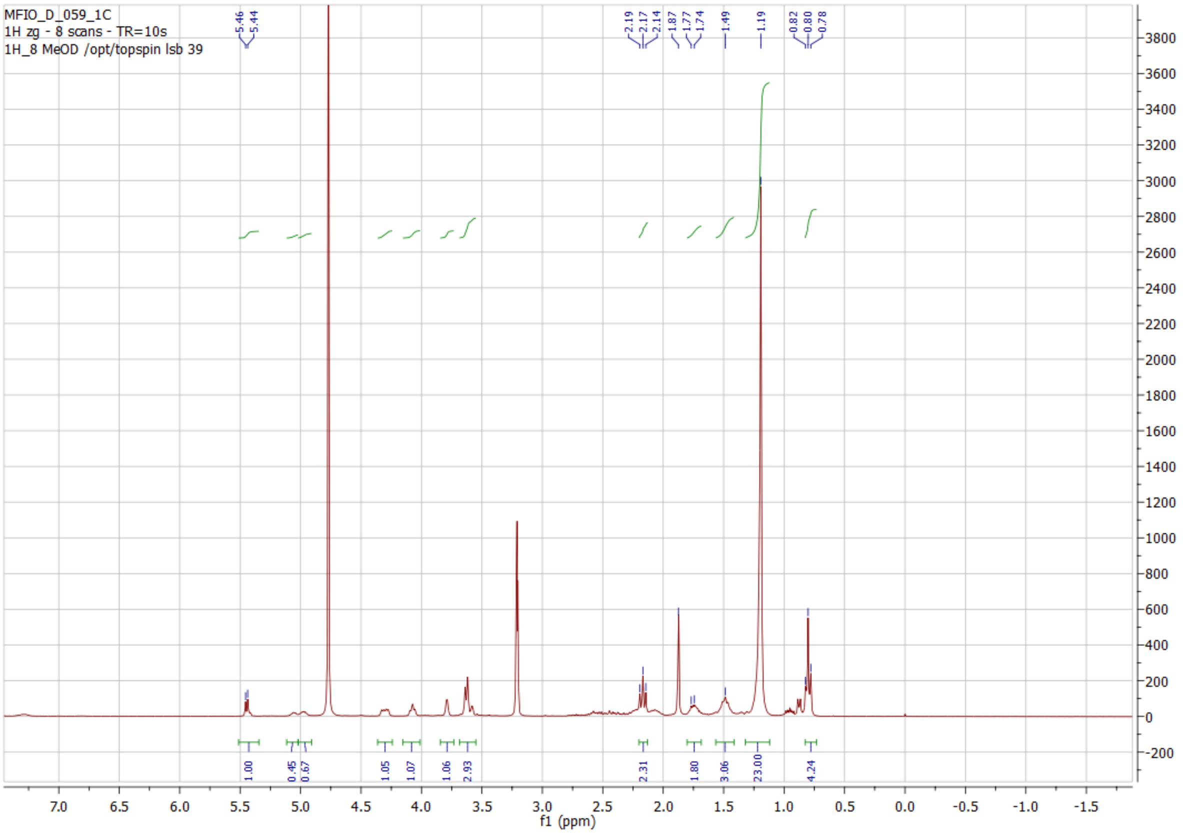
MFIO_D_059_1C
1H zg - 8 scans - TR=10s
1H_8 MeOD /opt/topspin lsb 39
f1 (ppm)

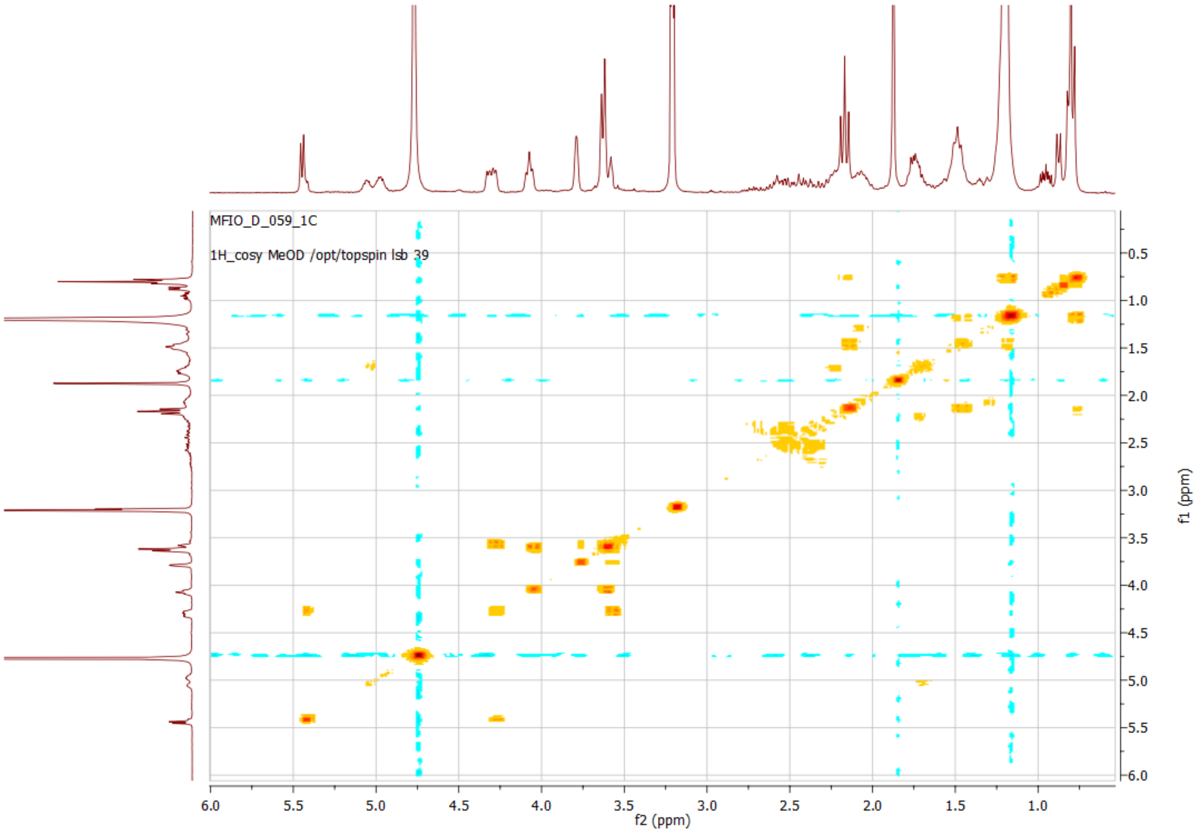
MFIO_D_059_1C
1H_cosy MeOD /opt/topspin lsb 39
f2 (ppm)
f1 (ppm)

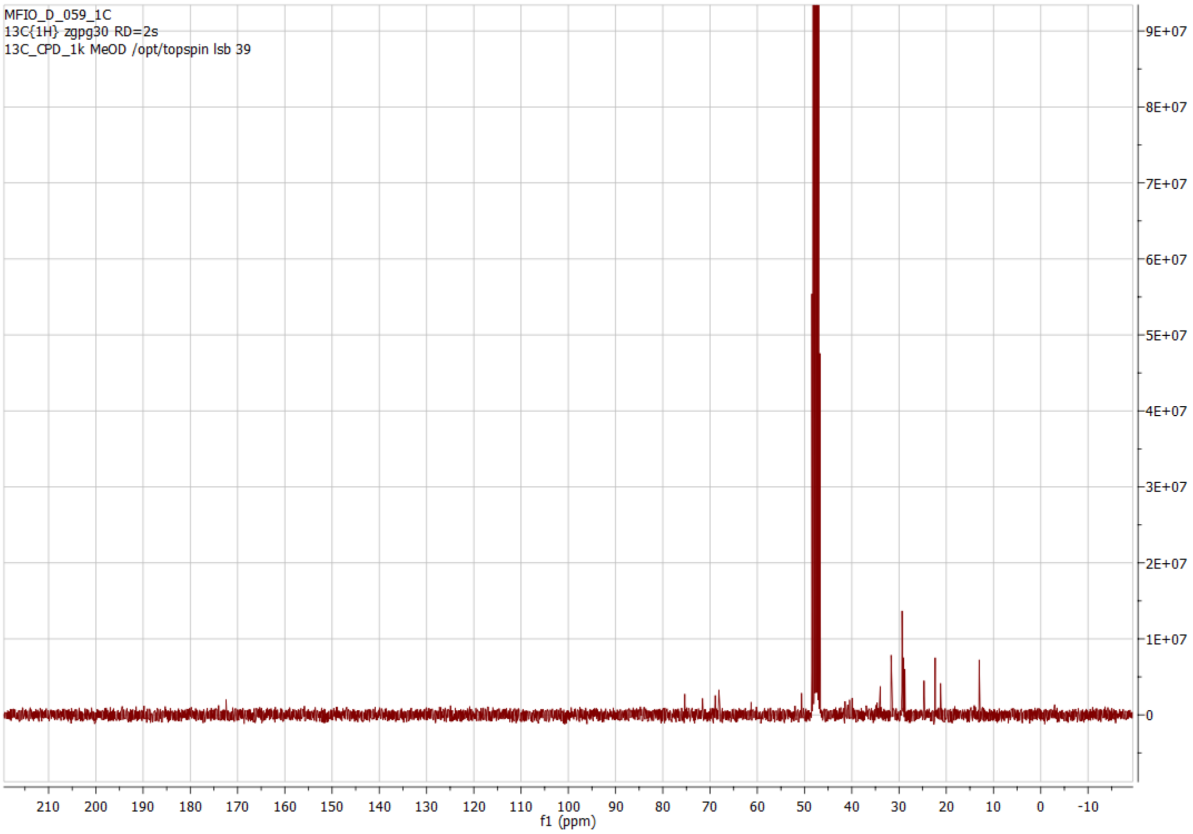

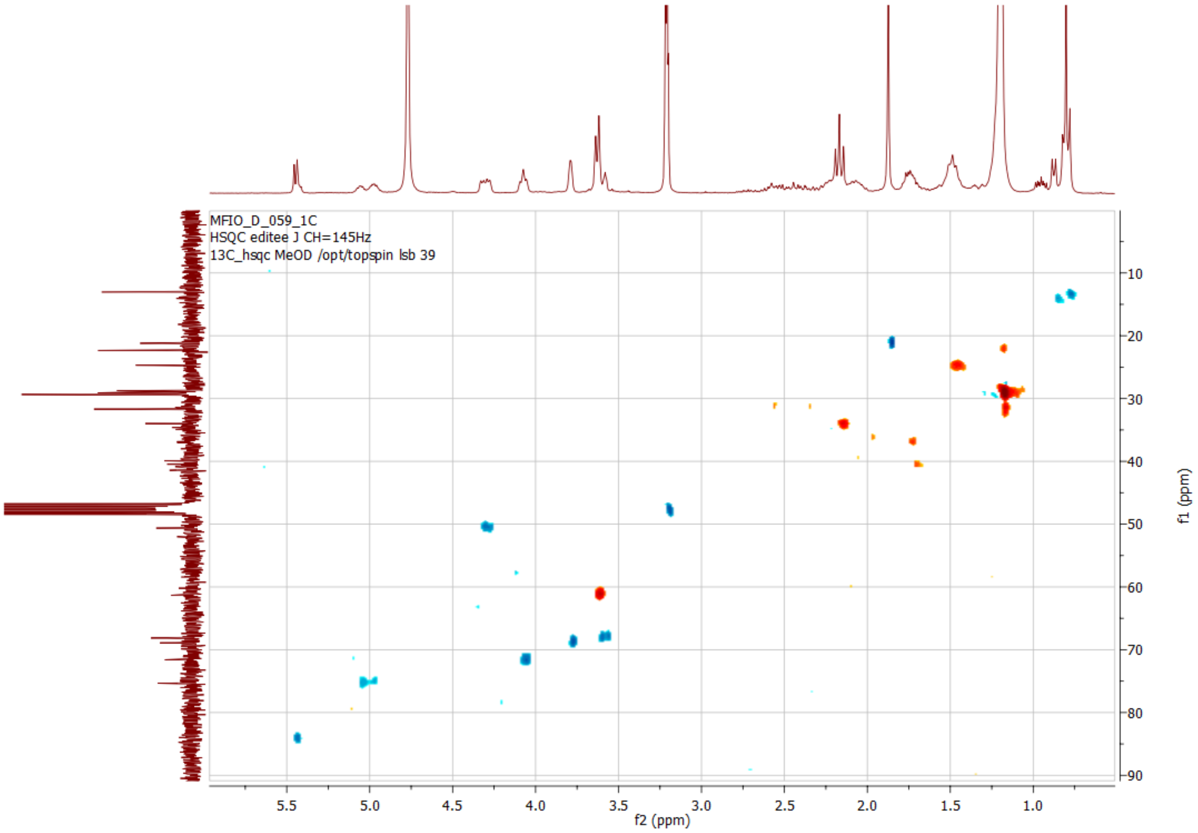

Figure S8-10 ^{1}H, g-COSY, ^{13}C and HSQC of compound **1c** in MeOD.
